# Synthesis of Isorhamnetin-3-*O*-Rhamnoside by a Three-Enzyme (Rhamnosyltransferase, Glycine Max Sucrose Synthase, UDP-Rhamnose Synthase) Cascade Using a UDP-Rhamnose Regeneration System

**DOI:** 10.3390/molecules24173042

**Published:** 2019-08-22

**Authors:** Anna Chen, Na Gu, Jianjun Pei, Erzheng Su, Xuguo Duan, Fuliang Cao, Linguo Zhao

**Affiliations:** 1 Co-Innovation Center for Sustainable Forestry in Southern China, Nanjing Forestry University, Nanjing 210037, China; 2 College of Chemical Engineering, Nanjing Forestry University, Nanjing 210037, China; 3 Jiangsu Key Lab of Biomass Based Green Fuels and Chemicals, Nanjing 210037, China

**Keywords:** rhamnosyltransferase, one-pot synthesis, isorhamnetin-3-*O*-rhamnoside, catalysis synergistic, UDP-rhamnose

## Abstract

Isorhamnetin-3-*O*-rhamnoside was synthesized by a highly efficient three-enzyme (rhamnosyltransferase, glycine max sucrose synthase and uridine diphosphate (UDP)-rhamnose synthase) cascade using a UDP-rhamnose regeneration system. The rhamnosyltransferase gene (78D1) from *Arabidopsis thaliana* was cloned, expressed, and characterized in *Escherichia coli*. The optimal activity was at pH 7.0 and 45 °C. The enzyme was stable over the pH range of 6.5 to 8.5 and had a 1.5-h half-life at 45 °C. The *V_max_* and *K_m_* for isorhamnetin were 0.646 U/mg and 181 μM, respectively. The optimal pH and temperature for synergistic catalysis were 7.5 and 25 °C, and the optimal concentration of substrates were assayed, respectively. The highest titer of isorhamnetin-3-*O*-rhamnoside production reached 231 mg/L with a corresponding molar conversion of 100%. Isorhamnetin-3-*O*-rhamnoside was purified and the cytotoxicity against HepG2, MCF-7, and A549 cells were evaluated. Therefore, an efficient method for isorhamnetin-3-*O*-rhamnoside production described herein could be widely used for the rhamnosylation of flavonoids.

## 1. Introduction

In nature, isorhamnetin glycosides, as the main derivatives of isorhamnetin, not only improve the solubility and stability of isorhamnetin but also exhibit anti-inflammatory [1], antinociceptive [2], and antioxidant activities [3]. Furthermore, isorhamnetin harboring different sugar residue results in diverse biological activities. Isorhamnetin-3-*O*-galactoside has been reported to have antithrombotic and profibrinolytic activities [4], while isorhamnetin-3-*O*-robinobioside can enhance antioxidant and antigenotoxic activity in human chronic myelogenous leukemia cell line K562 [5]. On the other hand, there was an interesting finding that the rhamnose moiety of steroidal alkaloids could affect the binding specificity to steroid receptors and triggering death of human hepatoma cell (Hep3B) by apoptosis [6]. In addition, some pharmacological studies also showed that many rhamnoside compounds possess significant pharmacological activities. For example, the myricetin-3-*O*-rhamnoside, isolated from *Myrtus communis* leaves, participates in the cellular defense system by regulating the oxidative stress response of cells and DNA damage repair [7]. Quercetin-7-*O*-rhamnoside has a strong inhibitory effect on the replication of the porcine diarrhea virus [8] and its structural analogue, quercetin-3-*O*-rhamnoside also shows a good inhibitory effect on the human influenza A/WS/33 virus [8,9]. As one kind of isorhamnetin glycosides, the isorhamnetin-3-*O*-rhamnoside only has been found in the *Cruciferae* [10], *Caesalpinia gilliesii* [11], *Formosan A. Purpurea* [12], and *Asturian cider apple* [13]. The isorhamnetin-3-*O*-rhamnoside was reported to exhibit moderate antioxidant activity [14] and the other biological properties remain to be studied.

The methods of extraction for the isorhamnetin-3-*O*-rhamnoside have been reported [10,11,12,13,14]. These methods are mainly based on the chemical reagents and plants resources and could be unfriendly to the environment. What’s worse, they are limited by the low concentration of product and complex secondary metabolites in the extracts. In comparison with chemical methods, enzymatic methods have many potentials for the isorhamnetin-3-*O*-rhamnoside production because of their high efficiency and environmental compatibility. The research about biological synthesis of isorhamnetin 3-*O*-glucoside using engineered glucosyltransferase has been reported [15]. The glycosyltransferases, which can transfer sugar molecules in their activated forms to acceptors, were considered to catalyze the most of glycosylation reactions in nature [16,17,18,19,20]. However, glycosylation reactions were limited by sugar donors. Sugar nucleotides are most commonly used as sugar donors and the uridine diphosphate (UDP) sugars are the largest group of nucleotide sugars. Nevertheless, most UDP sugars, especially UDP-rhamnose, which can be attached to small molecules such as secondary metabolites to improve their solubility, stability, and pharmacological properties [21,22,23,24], are expensive and hard to acquire. In our previous work, a cofactor self-sufficient UDP-rhamnose regeneration system has been established. This system can synthesize UDP-rhamnose from sucrose, Nicotinamide adenine dinucleotide (NAD^+^), and UDP by the catalysis of UDP-rhamnose synthase (VvRHM-NRS, cloned from *Vitis vinifera*) coupling with *Glycine max* sucrose synthase (GmSUS) and can be applied to the glycosylation of flavonoids by using the *A. thaliana* glycosyltransferase (78D1) [25].

**Figure 1:**
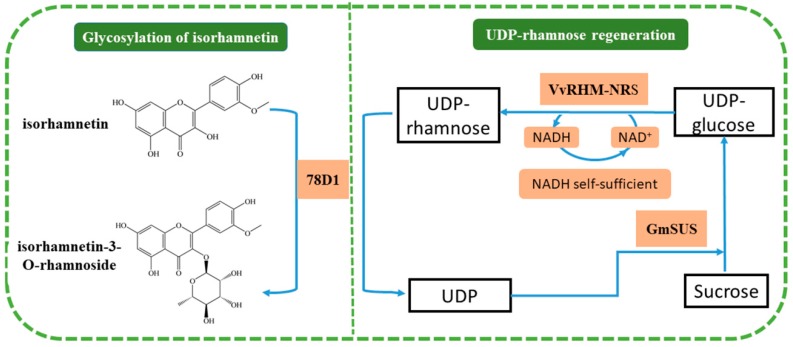
Schematic diagram of synergistic catalysis for isorhamnetin-3-*O*-rhamnoside by the combination of uridine diphosphate (UDP)-rhamnose regeneration and glycosylation of isorhamnetin.

In this paper, 78D1 was cloned, expressed, and characterized. Moreover, we have developed a process for the biosynthesis of isorhamnetin-3-*O*-rhamnoside by a three-enzyme cascade on the basis of the self-sufficient UDP-rhamnose regeneration system (Figure 1). Then, isorhamnetin-3-*O*-rhamnoside was prepared and used to evaluate the cytotoxicity against HepG2, MCF-7, and A549 cells.

## 2. Results and Discussion

### 2.1. Characterization and Purification of 78D1

Rhamnosyltransferase, which is widely found in nature, is involved in the production of secondary metabolites and plays an important role in the structural composition and various physiological functions of organisms. Some rhamnosyltransferases, which can catalyze the rhamnosylation of phenolic hydroxyl groups in flavonoids at different positions, were cloned and identified from different plants. For example, a flavonol 3-*O*-glucoside (1→6) rhamnosyltransferase GmF3G6″Rt was cloned from *soybean* [26], which can convert kaempferol 3-*O*-glucoside to kaempferol 3-*O*-rutinoside and utilize 3-*O*-glucosylated/galactosylated flavonols and UDP-rhamnose as substrates. Casas et al. [27] cloned the glycosyltransferase UGT91L1 from the *maize*, and the results of enzyme function identification showed that UGT91L1 could use isoorientin and UDP-rhamnose as substrates and convert them to rhamnosylisoorientin. However, most of these rhamnosyltransferases have not been characterized. Jones et al. [28] cloned 78D1 from *Arabidopsis thaliana* and confirmed that 78D1 catalyzes the transfer of rhamnose from UDP-rhamnose to the 3-OH position of quercetin and kaempferol, which means that 78D1 can be widely applied to the rhamnosylation of flavonoids. Thus, it is necessary to characterize the rhamnosyltransferase 78D1.

**Figure 2:**
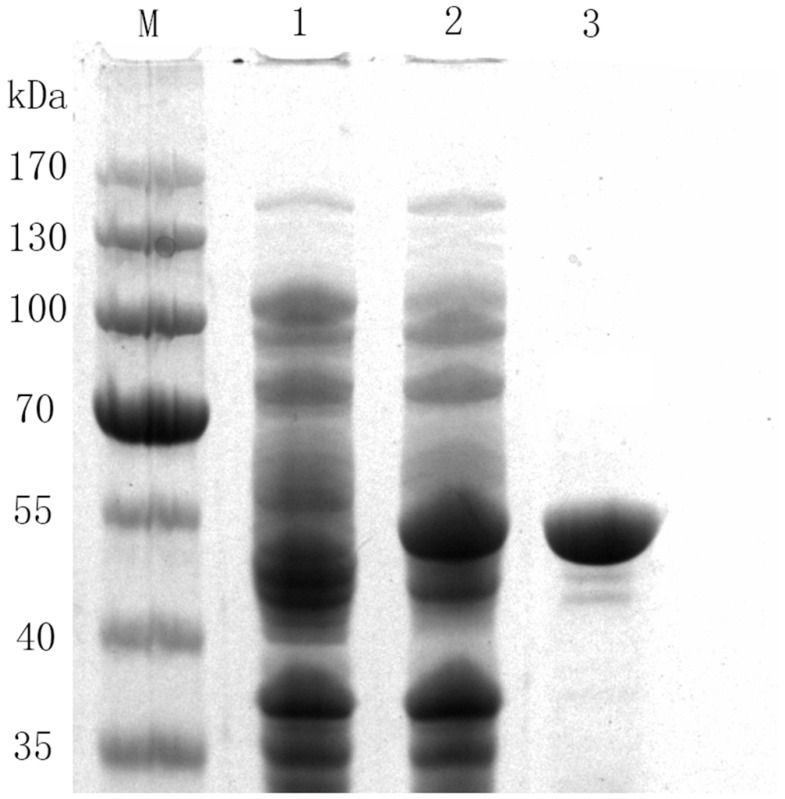
Polyacrylamide gel electrophoresis (SDS-PAGE) analysis of recombinant enzymes from *E. coli*. Lane 1: protein marker, lane 2: the crude extract of *E. coli BL21*, lane 3: the crude extract of 78D1, and lane 4: purified 78D1.

In order to improve the expression level of 78D1 in *E. coli*, we designed and optimized codons of 78D1 for the *E. coli* expression system. The recombinant 78D1 was expressed by adding 0.1 mM Isopropyl β-d-Thiogalactoside (IPTG) at 20 °C for approximately 12 h. Recombinant 78D1 was purified by Nickel affinity (Ni-NTA) column. The purification gave a single band on a 12% polyacrylamide gel electrophoresis (SDS-PAGE) gel and the molecular mass of the enzyme was less than 55 kDa without undesired bands (Figure 2), which was as similar as the theoretical molecular mass of the monomer (50019 Da).

**Figure 3:**
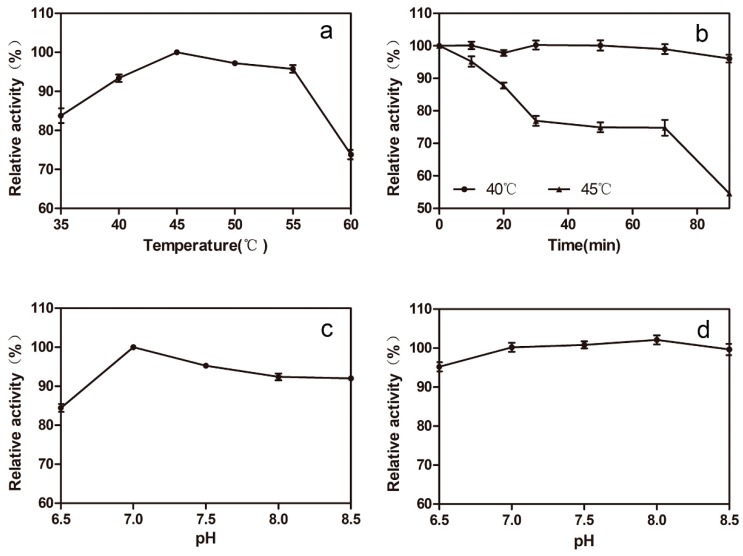
The effects of pH and temperature on the activity and stability of 78D1. (**a**) Effects of temperature on 78D1 activity. (**b**) The thermostability of 78D1. (**c**) Effects of pH on 78D1 activity. (**d**) Effects of pH on 78D1 stability. The initial activity was defined as 100%.

The biochemical properties of the 78D1 were characterized by using the purified recombinant 78D1. The optimal temperature of 78D1 is 45 °C (Figure 3a), and the enzyme activity was higher than 80% of the maximum activity when the temperature ranges from 35 to 55 °C. The thermostability of 78D1 was assayed and indicated that the residual activity of the enzyme was more than 95% after being incubated at 40 °C for 90 min, and more than 50% after being incubated at 45 °C for 90 min (Figure 3b). The optimal pH of 78D1 was 7.0 (Figure 3c), while its activity was higher than 80% of the maximum activity when the pH ranges from 6.5 to 8.5. The 78D1 showed good stability after being treated in 50 mM phosphate buffers of different pH levels at 4 °C for 24 h, and the residual activity was more than 95% (Figure 3d). Apparent *K_m_* and *V_max_* of 78D1 for isorhamnetin were 181 μM and 0.646 U/mg respectively, and the *V_max_/K_m_* value of 78D1 for isorhamnetin was 3.56 U/(mg·mM).

**Figure 4:**
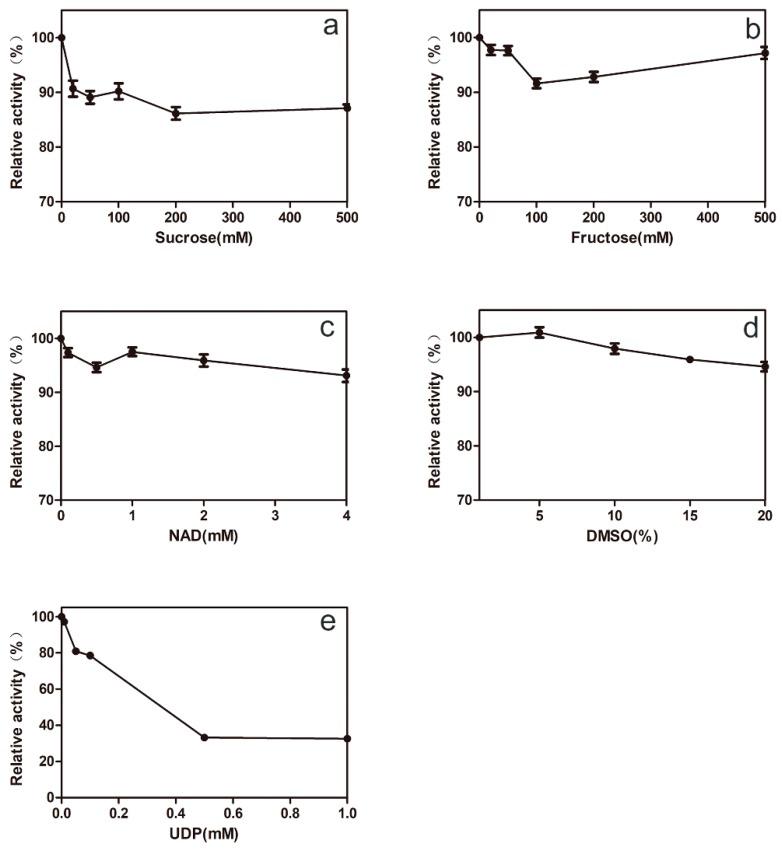
Effects of sucrose (**a**), fructose (**b**), NAD^+^ (**c**), DMSO (**d**), and UDP (**e**) on the activity of purified 78D1. The initial activity was defined as 100%.

It is important for 78D1 to adapt to the reaction conditions of synergistic catalysis. We investigated the effects of reaction substrates on the activity of 78D1. The effects of sucrose and fructose on the activity of 78D1 were not significant (Figure 4a,b), which suggested that 78D1 can adapt to the reaction conditions of GmSUS. The effect of NAD^+^ on 78D1 activity was not significant either (Figure 4c), which indicated that the enzyme can adapt to the reaction conditions of VvRHM-NRS. The enzyme activity was slightly increased when the concentration of Dimethyl sulfoxide (DMSO) was increased from 1% to 5% (Figure 4d). The enzyme activity was inhibited when the concentration of UDP was gradually increased (Figure 4e), which indicated that it is necessary to avoid the accumulation of UDP in the reaction system. These researchers suggest that recombinant 78D1 represents a potent candidate for the production of isorhamnetin-3-*O*-rhamnoside on the basis of the UDP-rhamnose regeneration system.

**Figure 5:**
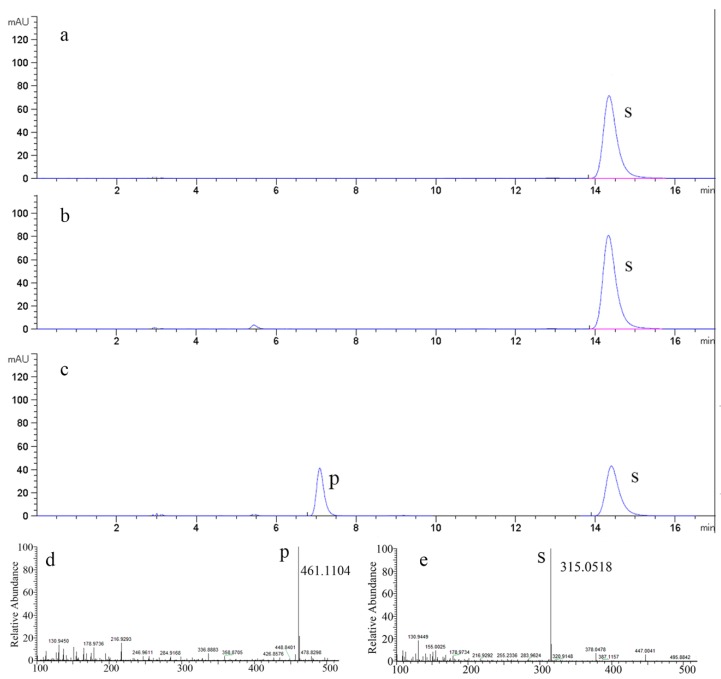
HPLC and LC/MS analysis of products synthesized from isorhamnetin. (**a**) HPLC analysis of the reaction system with 78D1 only. (**b**) HPLC analysis of the reaction system with 78D1 and GmSUS. (**c**) HPLC analysis of the reaction system with 78D1, GmSUS, and VvRHM. (**d**,**e**) LC/MS analysis of the product of the synergistic catalysis.

A certain amount of flavonoid product was synthesized by 78D1, GmSUS, and VvRHM-NRS (Figure 5c), while no flavonoid product was synthesized when the reaction mixture only had 78D1 or GmSUS and 78D1 (Figure 5a,b), which means that there is a synergistic reaction between three enzymes. A comparison of the *m/z* values of the molecular ion (M – H)^−^ of the product (461.1104) showed that the differences corresponded to a rhamnose residue in isorhamnetin (315.0518) (Figure 5d,e).

### 2.2. Optimizing the Ratio among 78D1, GmSUS, and VvRHM-NRS in a One-Pot Synthesis of Isorhamnetin-3-O-Rhamnoside

The effects of the ratio among 78D1, GmSUS, and VvRHM-NRS on isorhamnetin-3-*O*-rhamnoside production were determined (Table 1). Isorhamnetin-3-*O*-rhamnoside production increased 457% when the amount of VvRHM-NRS was increased from 125 to 500 μg/mL, whereas it only increased 183% and 280% when the amounts of GmSUS and 78D1 were raised from 8 to 88 μg/mL and from 9.5 to 95 μg/mL, respectively. This result indicates that the one-pot synthesis of isorhamnetin-3-*O*-rhamnoside catalyzed by VvRHM-NRS is the rate-limiting step in the present system. Isorhamnetin-3-*O*-rhamnoside production increased slightly when 78D1, GmSUS, or VvRHM-NRS continued to increase without changing the amounts of the other two enzymes. Thus, the optimal ratio among 78D1, GmSUS, and VvRHM-NRS was determined to be 57:50:375 (μg/mL).

### 2.3. Optimizing the Conditions of Synergistic Catalysis

**Figure 6:**
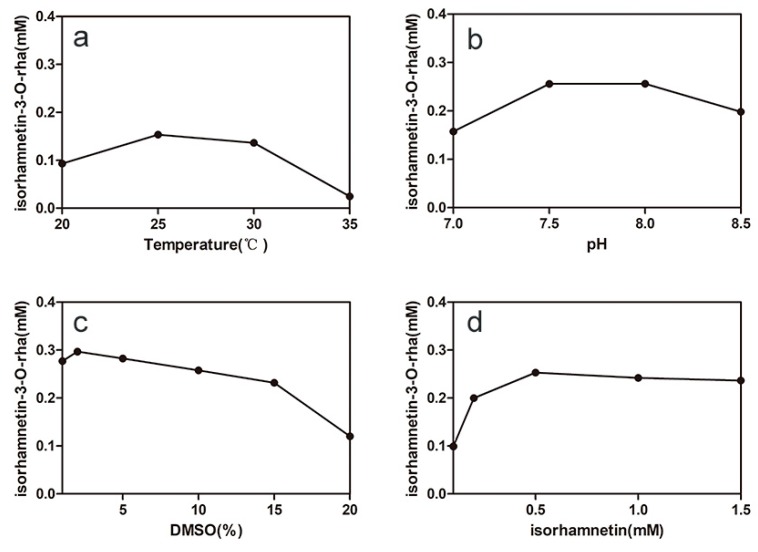
Optimization of bioconversion conditions for isorhamnetin-3-*O*-rhamnoside production by synergistic catalysis. Effects of temperature (**a**), pH (**b**), DMSO (**c**), and isorhamnetin (**d**) concentration on production. Values shown are the mean of duplicate experiments, and the variation about the mean was below 5%.

Temperature and pH are important factors in the one-pot synthesis of isorhamnetin-3-*O*-rhamnoside because of the effect on enzyme-specific activities and stabilities. The result showed that the optimal pH and temperature for synergistic catalysis were pH 7.5 and 25 °C (Figure 6a,b), respectively. Isorhamnetin-3-*O*-rhamnoside production was 16% of the maximum production at 35 °C because of the poor thermostability of VvRHM-NRS, and the lower temperature is more beneficial to sustaining the thermostabilities of the enzymes. Therefore, the temperature 25 °C was used for the following experiments.

The influence of DMSO concentration on the synergistic catalysis was studied (Figure 6c). The isorhamnetin solubility can be improved by DMSO to solve the problem that the poor solubility of isorhamnetin in the reaction mixture inhibits the activity of enzyme 78D1. The production was slightly increased when the concentration of DMSO was increased from 1% to 2%. When the concentration of DMSO exceeded 15%, isorhamnetin-3-*O*-rhamnoside production rapidly decreased. The production of isorhamnetin-3-*O*-rhamnoside increased as the concentration of isorhamnetin increased from 0.1 mM to 0.5 mM and the maximal production reached 0.25 mM in 15 h (Figure 6d).

### 2.4. Effects of Uridine Diphosphate (UDP), NAD^+^, and Sucrose on the One-Pot Synthesis of Isorhamnetin-3-O-rhamnoside

**Figure 7:**
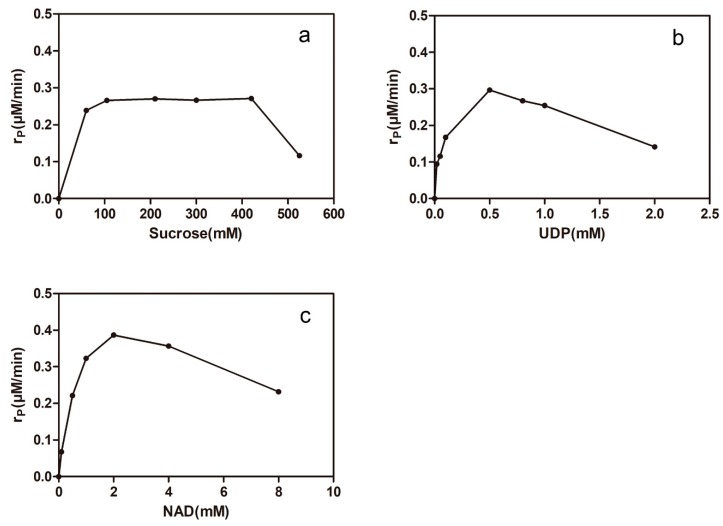
Isorhamnetin-3-*O*-rhamnoside production rate (r_p_) by synergistic catalysis as a function of (**a**) sucrose, (**b**) UDP, and (**c**) NAD^+^ concentrations. Values shown are the mean of duplicate experiments, and the variation about the mean was below 5%.

The isorhamnetin-3-*O*-rhamnoside production rate was measured when the initial concentrations of sucrose (60–525 mM; 0.5 mM UDP; 1 mM NAD^+^), UDP (0.02–2 mM; 300 mM sucrose; 1 mM NAD^+^), and NAD^+^ (0.1–8 mM; 0.5 mM UDP; 300 mM sucrose) were varied, respectively. The results are shown in Figure 7. The isorhamnetin-3-*O*-rhamnoside production rate was significantly decreased as the concentration of sucrose was increased from 420 mM to 525 mM (Figure 7a). UDP is a complex factor of synergistic catalysis, which is not only essential for GmSUS but is also a potent inhibitor of 78D1. The isorhamnetin-3-*O*-rhamnoside production rate was increased approximately three-fold when the concentration of UDP was increased from 20 to 500 μM and was decreased by half when the concentration of UDP exceeded 0.5 mM (Figure 7b). The isorhamnetin-3-*O*-rhamnoside production rate was also increased approximately six-fold when the concentration of NAD^+^ was increased from 0.1mM to 2 mM (Figure 7c). The production rate decreased by half when the concentration exceeded 2 mM because of the fact that NAD^+^ was also a potential inhibitor of the activity of VvRHM-NRS and high concentration of NAD^+^ could inhibit nicotinamide adenine dinucleotide (NADH) to serve as the cofactor of VvRHM-NRS. Based on the conversion efficiency, 400 mM sucrose, 0.5 mM UDP, and 2 mM NAD^+^ were used for the next experiments.

### 2.5. The Time Courses of Isorhamnetin-3-O-rhamnoside Production and Preparation

**Figure 8:**
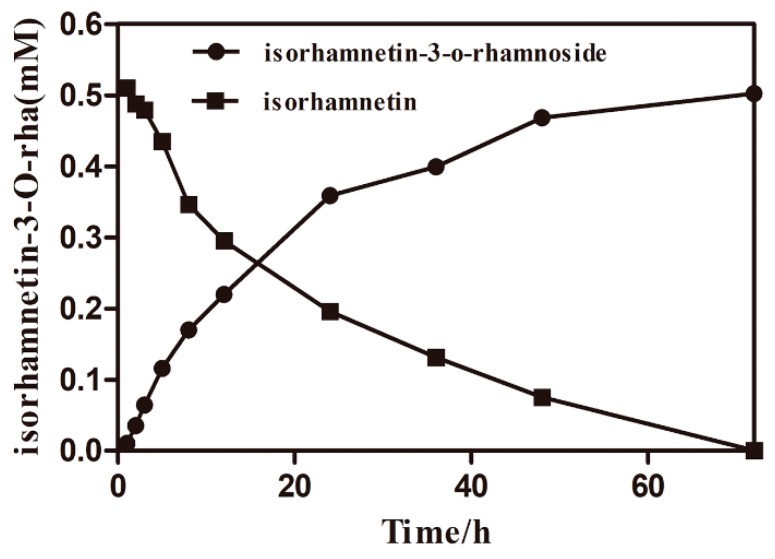
Time courses of isorhamnetin-3-*O*-rhamnoside production and isorhamnetin reduction in a synergistic reaction system. The circle represents isorhamnetin-3-*O*-rhamnoside. The triangle represents isorhamnetin.

On the basis of the consequence presented above, the optimal conversion conditions were determined and used for the production of isorhamnetin-3-*O*-rhamnoside. The specific productivity was 4.5 mg/L/h during the first hour after the initiation of synergistic catalysis (Figure 8). The specific productivity gradually increased as the reaction proceeded. The specific productivities were 9.86 mg/L/h over a reaction time of 1–3 h, 7.98 mg/L/h over a reaction time of 3–12 h, 5.36 mg/L/h over a reaction time of 12–24 h, 2.10 mg/L/h over a reaction time of 24−48 h, and 0.65 mg/L/h over a reaction time of 48–72 h. The main cause of the reduction in the specific productivity was the effect of product inhibition. After 72 h, 231 mg/L isorhamnetin-3-*O*-rhamnoside was produced with a corresponding molar conversion of 100%. After reparative liquid chromatography, rotary evaporation, and freeze-drying, isorhamnetin-3-*O*-rhamnoside was prepared from isorhamnetin in 89.8% overall yield and the final production of product was 50.1 mg. The structure of the product from synergistic catalysis was determined. The ^1^H and ^13^C-NMR spectra were recorded on a Bruker AVANCE IIII 400 spectrometer at 400 MHz (^1^H-NMR) and 100 MHz (^13^C-NMR) in DMSO-*d*_6_, with Me_4_Si as the internal standard. ^1^H-NMR (400 MHz, DMSO-*d_6_*) δ 7.43 (d, *J* = 2.0 Hz, 1H, 6′-H), 7.37 (dd, *J* = 8.3, 2.1 Hz, 1H, 5′-H), 6.93 (d, *J* = 8.3 Hz, 1H, 2′-H), 6.40 (s, 1H, 8-H), 6.18 (s, 1H, 6-H), 5.28 (d, *J* = 1.7 Hz, 1H, 1″-H), 4.95 (d, *J* = 4.4 Hz, 1H, 5″-H), 4.75 (d, *J* = 4.0 Hz, 1H, 2″-H), 4.63 (s, 1H, 3″-H), 3.96 (s, 1H, 4″-H), 3.84 (s, 3H, 3′-OCH3), 3.50 (d, *J* = 10.8 Hz, 2H, 3″, 4″-OH), 0.78 (d, *J* = 5.7 Hz, 3H, 2″-CH3). ^13^C-NMR (100 MHz, DMSO-*d_6_*) δ 177.32 (4-C), 172.96 (7-C), 156.61 (5-C), 149.78 (9-C), 147.26 (2-C), 133.98 (3-C), 122.48 (3′-C), 120.60 (4′-C), 115.46 (1′-C), 112.69 (6′-C), 104.04 (5′-C), 103.28 (2′-C), 101.68 (10-C), 99.26 (8-C), 94.07 (6-C), 72.51 (1″-C), 71.12 (5″-C), 70.03 (2″-C), 69.77 (3″-C), 63.06 (4″-C), 55.71 (3′-OCH3), 17.48 (2″-CH3) (Figures S1 and S2).The ^1^H-NMR spectrum was analyzed and compared to reference compounds^10^. The result confirmed that the catalysis synergistic product was isorhamnetin-3-*O*-rhamnoside. Obtained Isorhamnetin-3-*O*-rhamnoside thus meets commercial standards of quality and was used for the next research.

### 2.6. Evaluation of Cytotoxicity against HepG2, MCF-7, and A549 Cells

**Figure 9:**
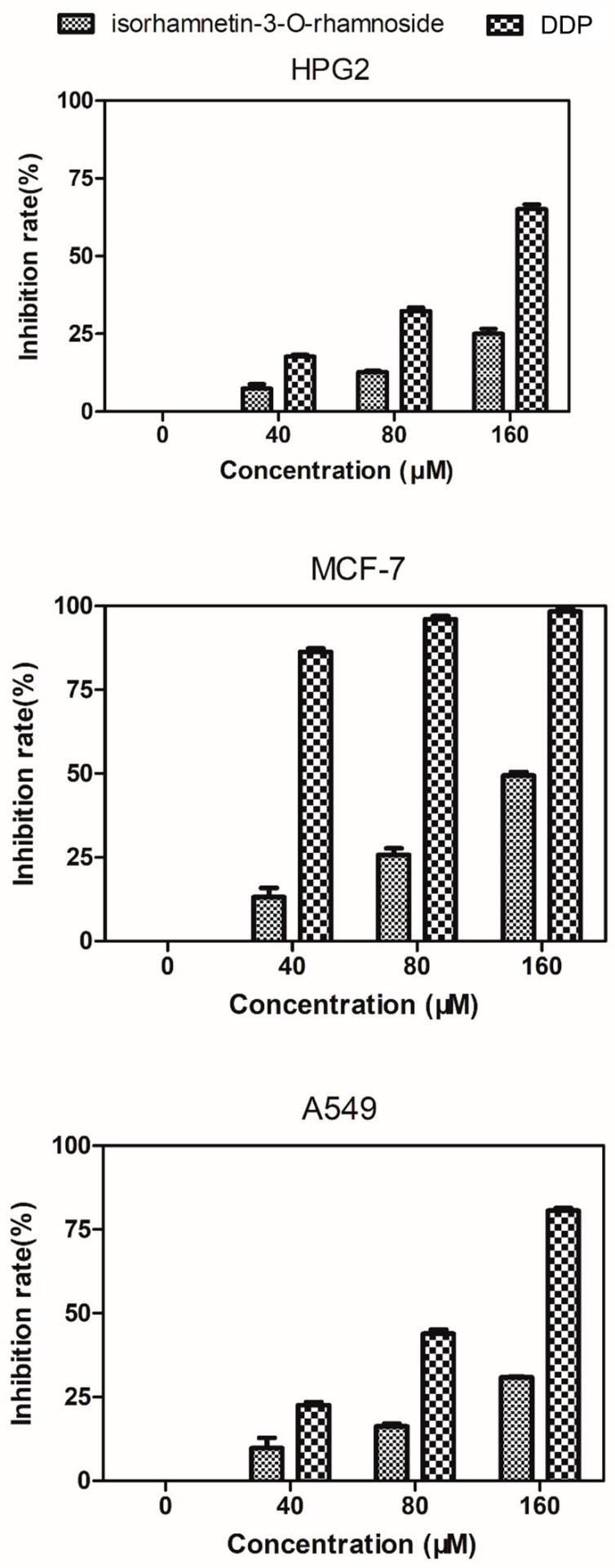
Isorhamnetin-3-*O*-rhamnoside evaluation of cytotoxicity against HepG2, MCF-7, and A549 cells. Various concentrations of Isorhamnetin-3-*O*-rhamnoside and DDP (0, 40, 80, or 160 μM) were added.

In this paper, we evaluated antiproliferative activities of isorhamnetin-3-*O*-rhamnoside and cisplatin (DDP) against HepG2, MCF-7, and A549 cells using the Thiazolyl Blue Tetrazolium Bromide (MTT) assay. The cell proliferation inhibition rates are shown in Figure 9. There is no inhibiting effect on the HepG2, MCF-7, and A549 cells when the isorhamnetin-3-*O*-rhamnoside and DPP do not exist. Moreover, the isorhamnetin-3-*O*-rhamnoside and DPP had lower inhibitory effects on human hepatoma cell line HepG2 and human lung cancer cell line A549. 160 μM isorhamnetin-3-*O*-rhamnoside showed strong inhibitory effects on human breast adenocarcinoma cell line MCF-7 proliferation (inhibition rate = 51%), though the same concentration DPP (positive control) showed the strongest inhibitory effects on human breast adenocarcinoma cell line MCF-7 proliferation (inhibition rate = 97%).

## 3. Materials and Methods

### 3.1. Strains, Plasmids, Media, and Chemicals

The plasmid propagated by *Escherichia coli* Top10 and recombinant enzymes were produced by *Escherichia coli* BL21 (DE3). PET-28a was purchased from Novagen (Darmstadt, Germany). The strains were cultured at 37 °C in Luria-Bertani (LB) medium supplemented with appropriate antibiotics. UDP and NAD^+^ were purchased from Sigma Chemical Co (St. Louis, MO, USA). UDP-rhamnose was purchased from Xiyuan Biotechnology Co (Shanghai, China). Isorhamnetin was purchased from MUST Bio-Technology (Chengdu, China).

### 3.2. Plasmid Construction

We designed and optimized codons of *78D1* for *E. coli* expression system. The *BamH*I and *EcoR*I sites were added to the 5′ and 3′ ends of the gene, respectively. The synthesized 78D1 was digested with *BamH*I and *EcoR*I and subcloned into the expression plasmid PET-28a at the *Bam H*I and *EcoR*I sites to create PET-28a-78D1.

### 3.3. Purification of Recombinant Enzymes

Recombinant enzymes GmSUS and VvRHM-NRS were prepared in our previous work [25]. The PET-28a-78D1 plasmid was introduced into *E.coli* BL21 (DE3) and induced to express by adding 0.1 mM isopropyl-β-d-thiogalactopyranoside (IPTG) at an OD600 of approximately 0.7. Then, the strain was incubated at 20 °C for approximately 24 h. Recombinant cells (1000 mL) were harvested by centrifugation at 5000× *g* for 10 min at 4 °C and washed twice with distilled water. The detailed procedures were performed according to the previous publication [29]. The recombinant 78D1 was purified by Ni^2+^-NTA affinity chromatography column and was examined by SDS-PAGE. The protein concentration was determined by the Bradford method using Albumin from bovine serum (BSA) as a standard.

### 3.4. Glycosyltransferase Activity

To determine the activity of 78D1, the reaction mixture was incubated at 35 °C for 30 min and contained 50 mM phosphate buffer (pH 7.5), excess UDP-rhamnose, 0.5 mM isorhamnetin, a certain amount of 78D1 in 100 μL. The reaction was stopped by adding 200 μL of methanol and was then assayed via high-performance liquid chromatography (HPLC). One unit of enzyme activity was defined as the amount of enzyme necessary to synthesize 1 μmol isorhamnetin-3-*O*-rhamnoside per min under the assay conditions.

The effects of temperature on the enzyme activity were determined over a temperature range of 35–60 °C in 50 mM phosphate buffer, pH 7.5. To determine the effect of temperature on the stability of 78D1, the enzyme in the 50 mM phosphate buffer (pH 7.5) was pre-incubated for various times at 40 °C and 45 °C in the absence of the substrate. The effects of pH on the enzyme activity were assayed over a pH range of 6.0–8.5 at 35 °C for 30 min using 50 mM phosphate buffer. To determine the effects of pH on the stability of 78D1, the enzyme in the 50 mM phosphate buffer (pH 6.5–8.5) was pre-incubated for 24 h at 4 °C in the absence of the substrate.

The effects of sucrose (0, 10, 50, 100, 200, and 500 mM), fructose (0, 10, 50, 100, and 200 mM), NAD^+^ (0, 0.1, 0.5, 1, 2, and 4 mM), dimethyl sulfoxide (DMSO) (1%, 5%, 10%, 15%, and 20% *v/v*), and UDP (0, 0.01, 0.05, 0.1, 0.5, 1, and 5 mM) on the 78D1 activity were assayed, respectively. The enzyme was incubated with different concentrations of substrates for 5 min at 35 °C before adding UDP-rhamnose to start the enzymatic reaction. The activity of 78D1 was determined as described above and the activity without pre-incubation was defined as 100%. The kinetic constant of 78D1 was determined by measuring the initial rates at different isorhamnetin concentrations under standard reaction condition.

### 3.5. Synergistic Catalysis

Reaction mixture containing 0.5 mM isorhamnetin, 300 mM sucrose, 1 mM NAD^+^, 0.5 mM UDP, 50 mM phosphate buffer (pH 7.5), 10% DMSO (*v/v*), rhamnosyltransferase 78D1, sucrose synthase GmSUS, and VvRHM-NRS in 100 μL was incubated for 15 h at 30 °C. 1 mM DTT was added to keep the stability and activity of VvRHM-NRS. The reaction was stopped by adding 200 μL of methanol and assayed via HPLC.

The effects of pH on the synergistic catalysis were assayed over a pH range of 7.0–8.5 at 30 °C for 15 h in 50 mM phosphate buffer. The effects of temperature on synergistic catalysis were determined over a temperature range of 25–35 °C in 50 mM phosphate buffer, pH 7.5. To optimize the conversion conditions, the concentrations of substrates and DMSO were varied separately: UDP (0–2 mM), sucrose (0–525 mM), NAD (0–8 mM), isorhamnetin (0–1.5 mM), and DMSO (1%–20%, *v/v*). To determine the optimal ratio among GmSUS, VvRHM-NRS, and 78D1, different ratios of enzymes were added into standard reaction mixtures.

### 3.6. Synthesis and Purification of Isorhamnetin-3-O-Rhamnoside

The substrate concentrations in the conversion were improved. The reaction mixture contained 0.5 mM isorhamnetin, 400 mM sucrose, 0.5 mM UDP, 2 mM NAD^+^, 50 mM phosphate buffer (pH 7.5), 2% DMSO (*v/v*), 57 μg/mL rhamnosyltransferase 78D1, 50 μg/mL sucrose synthase GmSUS, and 375 μg/mL VvRHM-NRS in 100 μL. The reaction volume was 250 mL and was incubated at 25 °C and 150 rpm in a thermomixer. The reaction was ended by adding methanol and harvested by centrifugation at 20,000× *g* for 10 min. The supernatant was applied to preparative liquid chromatography (SHIMADZU, Japan) and a C18 column with methanol (A) and distilled water (B) at the A/B ratio of 55:45. The product was collected and evaporated to remove the methanol, then the product was frozen to dryness and analyzed by HPLC and NMR.

### 3.7. HPLC, LC/MS Analysis and Structural Identification

HPLC analysis of isorhamnetin and isorhamnetin-3-*O*-rhamnoside (5,7-dihydroxy-2-(4-hydroxy-3-methoxyphenyl)-3-(((2*R*,3*S*,4*S*,5*R*)-3,4,5-trihydroxy-6-methyltetrahydro-2*H*-pyran-2-yl)oxy)-4*H*-chromen-4-one) was performed by using an HPLC 1200 system (Agilent, USA) and a C18 (250 × 4.6 mm; 5 μm) column with methanol (A) and distilled water (B) at A/B ratios of 55:45 for 17 min. The flow rate was 0.8 mL/min, and detection was performed by monitoring the absorbance at 368 nm. Liquid Chromatograph Mass Spectrometer (LC/MS) for isorhamnetin and isorhamnetin-3-*O*-rhamnoside were analyzed in an LTQ Orbitrap XL LC/MS in negative mode with an anion trap analyzer. The ion spray was operated at 25 Arb N_2_/min, 3.5 kV, and 300 °C. The ^1^H and ^13^C-NMR spectra were recorded on a Bruker AVANCE IIII 400 spectrometer at 400 MHz (^1^H-NMR) and 100 MHz (^13^C-NMR) in DMSO-*d*_6_, with Me_4_Si as the internal standard.

### 3.8. Cell Culture and Cytotoxicity Assay

Human hepatoma cell line HepG2, human breast cancer cell line MCF-7, and human lung cancer cell line A549 were purchased from the Cell Bank of the Chinese Academy of Sciences. HepG2, MCF-7, and A549 cells were grown in dulbecco’s modified eagle medium (DMEM) medium supplemented with 10% fetal bovine serum. All cells were kept in a humidified incubator with 5% CO_2_ and 95% air at 37 °C. Cells were routinely collected by centrifugation at 800× *g* for five minutes.

The effect of isorhamnetin-3-*O*-rhamnoside on the proliferation of tumor cells was tested by MTT assay. After trypan blue staining, 3 × 10^3^ tumor cells were inoculated into each well of a 96-well plate. After 6 h, different concentrations of isorhamnetin-3-*O*-rhamnoside and cisplatin (DPP) were added to each well, and then the plate was cultured at 37 °C in a 5% CO_2_ atmosphere for 72 h. MTT solution (20 μL, 4 mg/mL) was added to the culture medium. After 4 h, the plate was centrifuged at 1000× *g* for 5 min, and the supernatant was removed. DMSO (200 μL) was added to the wells and mixed until the precipitate completely dissolved. The absorbance at 540 nm was measured and recorded. All experiments were performed in at least four parallels and repeated three times. The percentage of cell proliferation inhibition was calculated using the following formula: Cell proliferation inhibition = (1 − OD_sample_/OD_control_) × 100%.

## 4. Conclusions

An enzymatic method for isorhamnetin-3-*O*-rhamnoside production has not been reported, and the product was seldom reported in other literatures. In conclusion, the rhamnosyltransferase gene (78D1) from *Arabidopsis thaliana* was cloned, expressed, and characterized in *Escherichia coli*. Furthermore, this paper described a novel and simple multienzyme method for the synthesis of isorhamnetin-3-*O*-rhamnoside with high efficiency. We have reported that the quercetin can be the substrate of the three-enzyme synergistic system [25]. This work confirmed that isorhamnetin can also be the substrate of the three-enzyme synergistic system. The result confirmed that the regenerate UDP-rhamnose system, which was reported in our previous work, can be applied to the rhamnosylation of flavonoids.

## Figures and Tables

**Table 1:** Effect of Enzyme Concentration on Product Yields of Isorhamnetin-3-*O*-rhamnoside ^a.^

Entry	78D1 (μg/mL)	GmSUS (μg/mL)	VvRHM-NRS (μg/mL)	Isorhamnetin-3-*O*-rhamnoside (mM)
1	95	8	250	0.024
2	95	16	250	0.034
3	95	32	250	0.039
4	95	88	250	0.044
5	9.5	50	250	0.015
6	19	50	250	0.025
7	38	50	250	0.033
8	95	50	250	0.042
9	57	50	125	0.014
10	57	50	188	0.017
11	57	50	250	0.041
12	57	50	375	0.059
13	57	50	500	0.064

^a^ The reaction mixture was incubated in 25 °C for 2 h. Values shown are the mean of duplicate experiments, and the variation about the mean was below 5%.

## Supplementary Materials

The following are available online, Figure S1: ^1^H-NMR(A) and ^13^C-NMR(B) spectra of isorhamnetin production by synergistic catalysis. Figure S2: The structural formula of isorhamnetin-3-O-rhamnoside.
