# Supplementary material for: Synthesis of Isorhamnetin-3-O-Rhamnoside by a Three-Enzyme (Rhamnosyltransferase, Glycine Max Sucrose Synthase, UDP-Rhamnose Synthase) Cascade Using a UDP-Rhamnose Regeneration System
Source: Molecules. 2019 Aug 22;24(17):3042. doi: 10.3390/molecules24173042 (PMC6749346; doi:10.3390/molecules24173042)
Supplement: Supplementary file 1 [file molecules-24-03042-s001.pdf]

SUPPORTING INFORMATION

Synthesis of isorhamnetin-3-O-rhamnoside by a three-enzym (rhamnosyltransferase, glycine max sucrose synthase, UDP-rhamnose synthase) cascade using a UDP-rhamnose regeneration system

Anna Chen[a,b], Na Gu[a,b], Jianjun Pei[b,c], Erzheng Su[a], Xuguo Duan[a], Fuliang Cao[a], Linguo Zhao[a,b,c*]

[a] Co-Innovation Center for Sustainable Forestry in Southern China, Nanjing Forestry University,
Nanjing, China;

[b] College of Chemical Engineering, Nanjing Forestry University, Nanjing, China;

[c] Jiangsu Key Lab of Biomass Based Green Fuels and Chemicals, Nanjing, China.

[*]Correspondence: lg.zhao@163.com; Phone: +86-025-85427962.

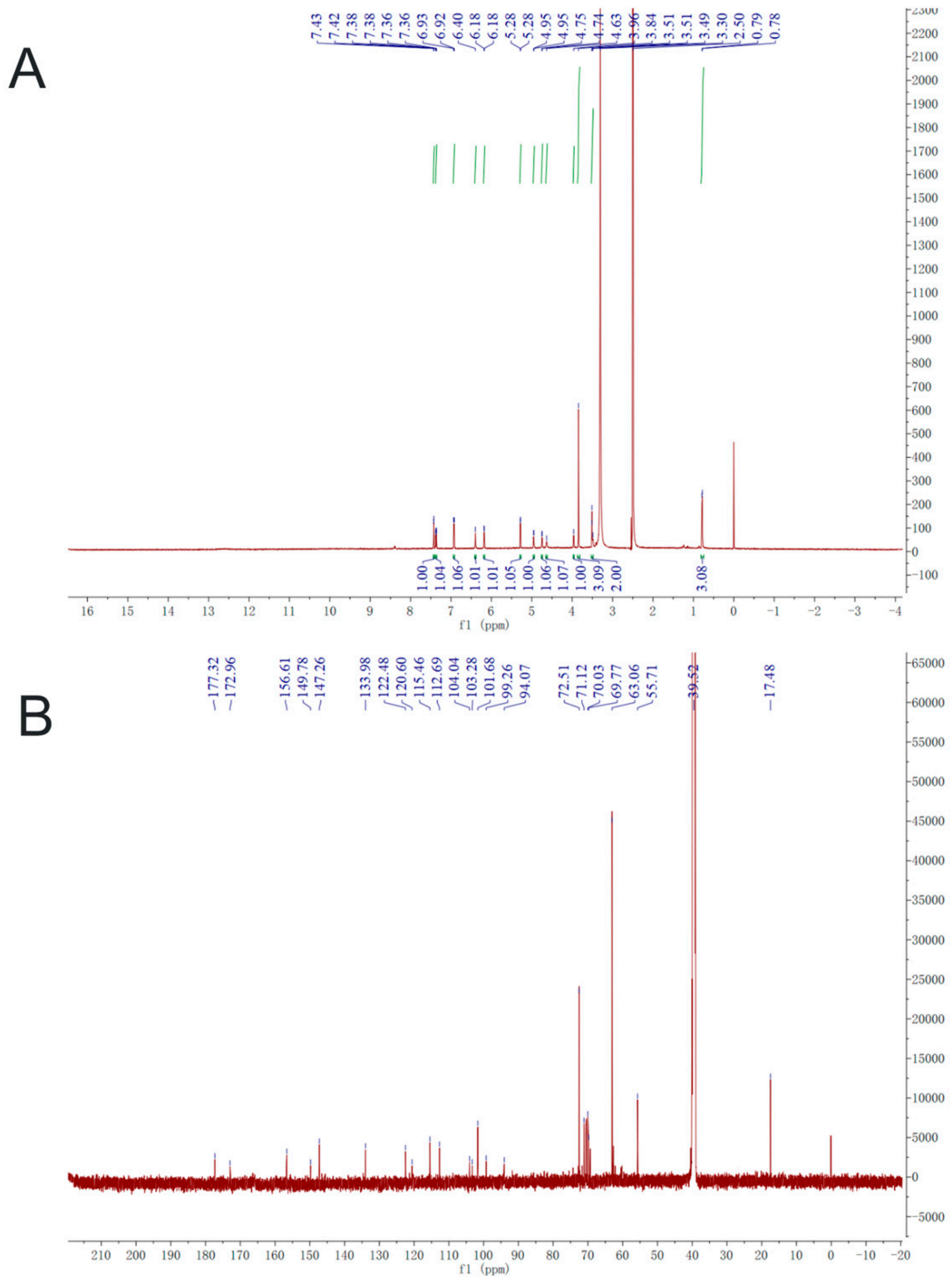

Fig S1. ^{1}H-NMR(A) and ^{13}C-NMR(B) spectra of isorhamnetin production by synergistic catalysis.

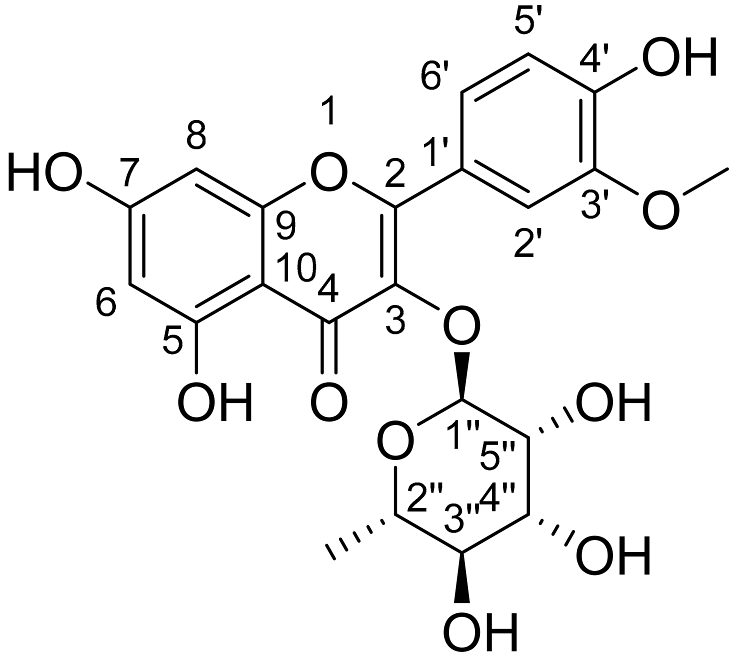

Fig S2. The structural formula of isorhamnetin-3-*O*-rhamnoside.
